# Pattern and outcome of chest injuries at Bugando Medical Centre in Northwestern Tanzania

**DOI:** 10.1186/1749-8090-6-7

**Published:** 2011-01-18

**Authors:** Monafisha K Lema, Phillipo L Chalya, Joseph B Mabula, William Mahalu

**Affiliations:** 1 Department of Surgery, Weill- Bugando University College of Health Sciences, Mwanza, Tanzania

## Abstract

**Background:**

Chest injuries constitute a continuing challenge to the trauma or general surgeon practicing in developing countries. This study was conducted to outline the etiological spectrum, injury patterns and short term outcome of these injuries in our setting.

**Patients and methods:**

This was a prospective study involving chest injury patients admitted to Bugando Medical Centre over a six-month period from November 2009 to April 2010 inclusive.

**Results:**

A total of 150 chest injury patients were studied. Males outnumbered females by a ratio of 3.8:1. Their ages ranged from 1 to 80 years (mean = 32.17 years). The majority of patients (72.7%) sustained blunt injuries. Road traffic crush was the most common cause of injuries affecting 50.7% of patients. Chest wall wounds, hemothorax and rib fractures were the most common type of injuries accounting for 30.0%, 21.3% and 20.7% respectively. Associated injuries were noted in 56.0% of patients and head/neck (33.3%) and musculoskeletal regions (26.7%) were commonly affected. The majority of patients (55.3%) were treated successfully with non-operative approach. Underwater seal drainage was performed in 39 patients (19.3%). One patient (0.7%) underwent thoracotomy due to hemopericardium. Thirty nine patients (26.0%) had complications of which wound sepsis (14.7%) and complications of long bone fractures (12.0%) were the most common complications. The mean LOS was 13.17 days and mortality rate was 3.3%. Using multivariate logistic regression analysis, associated injuries, the type of injury, trauma scores (ISS, RTS and PTS) were found to be significant predictors of the LOS (*P *< 0.001), whereas mortality was significantly associated with pre-morbid illness, associated injuries, trauma scores (ISS, RTS and PTS), the need for ICU admission and the presence of complications (*P *< 0.001).

**Conclusion:**

Chest injuries resulting from RTCs remain a major public health problem in this part of Tanzania. Urgent preventive measures targeting at reducing the occurrence of RTCs is necessary to reduce the incidence of chest injuries in this region.

## Background

Trauma continues to be an enormous public health problem worldwide and it is associated with high morbidity and mortality both in developed and developing countries [1]. Trauma is reported to be the leading cause of death, hospitalization, and long-term disabilities in the first four decades of life.

Globally, 10% of all trauma admissions result from chest injuries and 25% of trauma-related deaths are attributable to chest injuries [2,3].

In Tanzania, trauma including chest injuries continues to be one of the leading causes of morbidity among the young and old with an estimated mortality of 40% [4]. In Bugando Medical Centre, chest trauma has been commonest cause of surgical admission and contributes significantly to high morbidly and mortality [5].

The causes and pattern of chest injuries have been reported in literature to vary from one part of the world to another partly because of variations in infrastructure, civil violence, wars and crime. Road traffic crushes (RTCs) are the commonest cause of chest injuries in civilian practice accounting for up to 70% in some series [6,7]. With increasing use of firearms, arrows and spears the incidence of penetrating chest injuries increased in civil society [8]. They are often associated with other extra-thoracic injuries particularly to the abdomen and long bones [8,9].

Studies have shown that most chest injuries can be treated by non-surgical approach with relatively simple methods, such as tube thoracostomy, appropriate analgesics management, and good pulmonary toilet [10,11]. The accurate identification of a patient at high risk for major chest injuries is necessary to avoid delays that may lead to significant morbidity and mortality [12]. Aggressive management of the chest trauma along with prompt treatment of associated injuries is essential for optimal patient outcome [13].The majority of chest injuries are preventable. A clearer understanding of the causes, injury patterns and outcome of these patients is essential for establishment of prevention strategies as well as treatment protocols [14]. Such data is lacking in our environment as there is no local study which has been done.

This study was conducted in our setting to describe our own experience in the management of chest injuries, outlining the etiological spectrum, injury patterns and outcome of chest injuries in our local setting. The study results will provide basis for planning of prevention strategies and establishment of treatment protocols.

## Patients and Methods

### Study design and Setting

This was a prospective study which was conducted at the Accident and Emergency (A&E) department and surgical wards of Bugando Medical Centre (BMC) over a six-month period from November 2009 to April 2010 inclusive. Bugando Medical Centre is a referral, consultancy and teaching hospital for Weil Bugando University College of Health Sciences (WBUCHS) and other paramedics and it is located in Mwanza city in the northwestern part of Tanzania. It is situated along the shore of Lake Victoria and has a bed capacity of 1000. BMC is one of the four largest referral hospitals in the country and serves as a referral centre for tertiary specialist care for a catchment population of approximately 13 million people from Mwanza, Mara, Kagera, Shinyanga, Tabora and Kigoma.

Trauma patients are first seen at the A & E department where the surgical team does primary and secondary surveys. The surgical team comprising of intern doctors, Registrar [Medical Officers], Resident [Postgraduate student in Surgery] and Surgeon. From the A & E department these patients are admitted in respective surgical wards after definitive treatment either in operating theatre or at the A & E department.

### Study subjects

The study population included all chest injury patients of all age groups and both genders admitted to the surgical wards of BMC during the study period. Unconscious patients without next of kin to consent and give information and those who were still in the ward at the end of study period were excluded from the study. Patients who died before complete assessment and those who absconded against medical advice were also excluded. Recruitment of patient to participate in the study was done at the A&E department after primary and secondary surveys done by the admitting surgical team. Patients were screened for inclusion criteria and those who met the inclusion criteria were offered explanations about the study and requested to consent before being enrolled into the study. The diagnosis of chest injury was made by clinical history, physical examination and abnormal chest radiographs at the accident and emergency department. Chest injuries were considered as both blunt and penetrating affecting the chest wall and the contents of the thorax e.g. pleura, lungs, lower respiratory tract, esophagus, heart and great vessels. All recruited patients were managed according to advanced trauma life support [ATLS].

Associated injuries were managed appropriately according to type of injury. The authors ensured that the study patients were receiving the appropriate treatment and supportive care as prescribed by the surgeon. Patients were followed up till discharge or death. The length of hospital stay and mortality as measures of outcome of chest injury patients was recorded at the end of follow up period. The minimum follow-up period was thirty days. Data was collected using a pre-tested coded questionnaire. Data administered in the questionnaire included; demographic characteristics (e.g. age, sex), circumstances of injury, characteristics of injury causes, management, complications, Length of Hospital Stay (LOS) and mortality.

### Data collection and analysis

Data collected was entered into a computer and analyzed using SPSS software version 11.5 with help of a medical statistician. Data was summarized in form of proportions and frequency tables for categorical variables. Means, median, mode, standard deviation and histograms were used to summarize continuous variables. Chi-square test was used to test for significance of associations between the predictor and outcome variables in the categorical variables. Student t-test was used to test for significance of associations between the predictor and outcome variables in the continuous variables. Multivariate logistic regression analysis was used to determine predictor variables that are associated with outcome. Significance was defined as a *p-*value of less than 0.05.

### Ethical consideration

Ethical approval to conduct the study was obtained from the WBUCHS/BMC joint institutional ethic review committee before the commencement of the study. Informed consent was sought from each patient before recruitment into the study.

## Results

A total number of 150 chest injury patients were studied. Their ages ranged from 1 to 80 years with a mean of 32.17 years and standard deviation of 14.74. The median and the mode were 30.00 and 22.00 years respectively. The peak age incidence was 21-30 years

There were 119 (79.3%) males and 31 (20.7%) females with the male to female ratio of 3.8:1 with a male predominance in each age group (Table 1).

**Table 1:** Age group distribution according to sex

Age Group	Males	Females	Total
0-10	7(4.6%)	5(3.4%)	12(8.0%)
11-20	8(5.3%)	4(2.7%)	12(8.0%)
21-30	47(31.3%)	7(4.6%)	54(36.0%)
31-40	29(19.3%)	9(6.0%)	38(25.3%)
41-50	17(11.3%)	2(1.3%)	19(12.6%)
51-60	9(6.0%)	-	9(6.0%)
61-70	-	3(2.0%)	3(2.0%)
71-80	2(1.3%)	1(0.7%)	3(2.0%)

Total	**119(79.3%)**	**31(20.7%)**	**150(100%)**

Six patients (4.0%) presented with history of pre-morbid. There was significant association between premorbid illness and mortality (P = 0.047) but not LOS (P = 0.925). The majority of patients (95; 63.3%) were injured during the day. One hundred and ten patients (73.3%) were attended within 24 hours of the injury. The remaining forty patients (26.7%) were attended more than 24 hours since the injury occurred. Timing in seeking medical care did not significantly influence both LOS (P = 0.095) and mortality (P = 0.089).The majority of patients 109(72.7%) sustained blunt injuries. The remaining patients had either penetrating injuries in 40 patients (26.7%) or combined (blunt and penetrating) injuries in 1 (0.6%). The mechanism of injury was not significantly associated with LOS (P > 0.05) and mortality (P > 0.05).Road traffic accident was the most common cause of injuries affecting 76(50.7%) of patients (Table 2).

**Table 2:** Distribution of study population according to the cause of injury

Cause of injury	Number of patients	Percentage
Road traffic accident	76	50.7
Assault	42	28.0
Fall	24	16.0
Sport injuries	2	1.3
Others	6	4.0

**Total**	**150**	**100**

In this study, the cause of injury did not significantly affect the outcome of chest injury patients in terms of either LOS (P > 0.05) or mortality (P > 0.05. Thirty two patients (21.3%) received prehospital care. There was statistically significant association between prehospital treatment and either LOS (P = 0.001) and mortality (P = 0.001). Chest wall wounds, hemothorax and rib fractures were the most common type of injuries accounting for 30.0%, 21.3% and 20.7% respectively (Table 3).

**Table 3:** Type of injuries

Types of injury	Number of patients	Percentages
Chest wall wounds	45	30.0
Rib fractures	31	20.7
Flail chest	1	0.7
Clavicular fractures	4	2.7
Scapular fractures	7	4.7
Thoracic spine injury	18	12.0
Haemothorax	32	21.3
Pneumothorax	2	1.3
Pneumoheamothorax	11	7.3
Lung laceration	1	0.7
Hemopericardium	1	0.7
Esophageal injury	1	0.7
Acute Respiratory Distress Syndrome	3	2.0

The type of injury; (soft tissue thoracic trauma, rib fractures, hemothorax) significantly influenced the LOS (P < 0.05) but not mortality (P > 0.05). Associated extra-thoracic injuries were noted in 56.0% of patients and head/neck (33.3%) and musculoskeletal regions (26.7%) were commonly affected (Table 4).

**Table 4:** Distribution of study population according to associated injuries

Associated injuries	Number of patients	Percentage
Head/neck injuries	50	33.3
Abdominal injuries	8	5.3
Pelvic injuries	5	3.3
Musculoskeletal injuries	40	26.7
Multiple injuries	1	0.7

The mean Injury severity score (ISS), Revised trauma score (RTS) and Pediatric trauma score (PTS) were 7.41, 7.61 and 9.50 respectively and were statistically significantly associated with both LOS and morbidity (P < 0.05). All the patients had chest radiographs done; the commonest abnormal findings were hemothorax 32 (21.3%) and rib fractures 31(20.7%). Radiographs of limbs were done in 40 patients (26.7%) and detected fractures in 27 patients (18.0%). Abdominal ultrasound was done in eight patients and abnormal findings were detected in 3 patients (splenic rupture in 2 patients and haemoperitoneum alone in one patient). CT scan of the skull and brain was done in 31 patients (20.7%) and detected abnormality in 23 patients (15.3%) mainly brain edema in 14 patients ( 9.3%), skull fractures in 11 patients ( 7.3%) and space occupying lesions (epidural, subdural, subarachnoid and intra-cerebral hematoma) in 8 patients (5.3%). Out of 150 patients, 83 patients (55.3%) were treated by non-operative approach with analgesics, antibiotics and tetanus prophylaxis. Underwater seal drainage was performed in 29 patients (19.3%) in patients who had hemothorax, pneumothorax and haemopneumothorax and the average duration of drainage was 8.4 days. One patient (0.7%) underwent thoracotomy due to hemopericardium. Surgical treatment for associated injuries is shown in table 5.

**Table 5:** Treatment modalities of the associated injuries

Treatment modality	Frequency	Percentage
Wound debridement	44	29.3
Closed reduction of factures	23	15.3
Open Reduction & Internal Fixation (ORIF)	6	4.0
Exploratory laparotomy	3	2.0
- Spleenectomy	2	
- Repair of perforated bowels	1	

Craniotomy + burr holes	12	8.0
Skull fracture elevation	3	2.0

Thirteen patients (8.7%) were admitted in the ICU. The mean LOS in the ICU was 7.34 days.

The need for ICU admission was found to be significantly associated with mortality (P = 0.021) Thirty nine patients (26.0%) had complications. (Table 6).

**Table 6:** Distribution of study population according to complications

Complications	Frequency	Percentage
Wound sepsis	22	14.7
Complications of fractures	18	12.0
Post concussion syndrome	7	4.7
Pneumonia	5	3.3
Intra-abdominal complications	5	3.3
Empyema thoracis	4	2.7
Neurological deficit	4	2.7
Acute respiratory distress syndrome	1	0.7

The presence of complications was found to be significantly associated with mortality (P = 0.001) but not with LOS (P = 0.067)

### Outcome of treatment

The overall length of hospital stay ranged from 1 day to 120 days (mean =13.17 days). The LOS for non-survivors ranged from 1 day to 14 days (mean = 4.43 days). Table 7 shows multivariate logistic regression analysis for LOS.

**Table 7:** Multivariate logistics regression analysis for LOS

Independent variable	Odds ratio	95% Confidence Interval	p-value
Associated injuries	5.6	1.3-94.8	0.003
The type of injury	13.6	3.3-24.3	0.001
Injury Severity Score	2.6	0.9-8.7	0.010
Revised Trauma Score	12.3	5.6-28.7	0.001
Pediatric Trauma Score	11.2	4.2-23.8	0.012

In this study, seven patients died giving a mortality rate of 4.7%.Table 8 shows multivariate logistic regression analysis for mortality.

**Table 8:** Multivariate logistic regression analysis for mortality

Independent variable	Odds ratio	95% Confidence Interval	*p*-value
Pre-morbid illness	2.3	1.2-97.1	0.000
Associated injuries	15.1	7.5-20.9	0.001
The need for ICU admission	13.9	8.2-67.1	0.013
Injury Severity Score	6.6	1.9-8.9	0.020
Revised Trauma Score	17.3	5.2-26.7	0.011
Paediatric Trauma Score	15.2	4.2-43.6	0.015
Presence of complications	12.9	6.8-34.1	0.000

## Discussion

In this study, most of our patients were youth in their most productive years and showed a male preponderance. Similar demographic observation was also reported by other authors [8,9,15,16]. The reason for male predominance among chest injury patients in this age group is probably that males are more mobile with active participation in high risk taking activities. Identification of risk taking behavior among trauma patients has potential significance for the prevention of injuries.

The presence of pre-morbid illness has been reported to have an effect on the outcome of chest trauma patients [17,18]. In this study, pre-morbid illness was found to be significantly associated with mortality but not LOS.

Knowing the time of injury in trauma patient is important for prevention strategies and has an impact on the outcome. We noted that the majority of patients who arrived during night hours had poor prognosis compared to day arrivals. This can be explained by the fact that during night hours the majority of the senior surgical and auxiliary staff, whom we found to be pivotal in the diagnosis and management of chest injuries, were unlikely to be present unless called for difficult cases. In our resource-limited setting, where staff shortage is a challenging problem, re-distribution of the few staff available needs to be designed to address the problem.

Despite the fact that timing of medical care did not significantly affect the outcome of our patients in term of LOS and mortality, the author of the present study still believe that delay in seeking medical care still contributes significantly to high morbidity and mortality among chest injury patients. Early recognition and treatment of these injuries appear to reduce mortality and morbidity associated with the disease.

Most patients in this study sustained blunt chest injuries, which is comparable with other studies [19-21] but in contrast with a Nigerian study [15] in which penetrating chest injuries was the most common mechanism of injury. The high incidence of blunt chest injuries in this study is explained by the fact that those patients who had blunt injuries were mostly involved in road traffic crush another common feature of increased motorization in this environment.

Road traffic crush (RTC) remains a leading cause of trauma and admissions to the accidents and emergency units of most hospitals in Tanzania and contributing significantly to high morbidity and mortality [22]. In this study, RTCs were the most common cause of injuries and the majority of these were due to motorcycle accidents, an emerging popular mode of commercial transportation in Mwanza City, and the victims were passengers, cyclists and pedestrians. Findings from this study calls for urgent interventions targeting at reducing the occurrence of RTCs and subsequently reduce the incidence of these injuries in this region.

The management of patients with chest injuries has several important elements: adequate pre-hospital care, rapid transport to a specialized centre, complex in-hospital care and rehabilitation. The prehospital phase plays a vital role in determining the final outcome of treatment when done appropriately and contributes significantly to reducing morbidity and mortality [23]. In the present study, prehospital treatment was reported in only 21.3% of chest injury patients which is in agreement with a study that was done in Ethiopia [15]. Lack of advanced pre-hospital care in Mwanza city is the major challenge in providing care for trauma patients and have contributed significantly to poor outcome of these patients due to delay in definitive management.

The type of injuries in this study is comparable with what is reported in other studies in Nigeria [15,24] and Ethiopia [16]. In the present study, the type of injury significantly affected the LOS but not mortality. This finding reflects the low mortality rate among chest injury patients in this study.

The pattern of associated extra-thoracic injuries in this study is in agreement with findings from other studies done elsewhere [15,25,26]. The presence of associated injuries is an important determinant of the outcome of chest injury patients. Associated injuries increase the risk of complications in patients with chest injuries. Early recognition and treatment of associated extra-thoracic injuries is important in order to reduce mortality and morbidity associated with chest injuries [21].

In our study, all the patients had chest radiographs done. This is in agreement with other studies done elsewhere [15,16]. Ultrasound of the chest has been reported to be an important diagnostic tool in the diagnosis of haemopneumothorax, in patients where x-rays are not possible, as in unconscious patients and having pelvis or spine injury. Moreover, ultrasound also helps in differentiating between hemothorax and pulmonary contusion [27]. Atri *et al *[28] highlighted that ultrasound chest had a sensitivity of 94.6% for detecting pulmonary contusions and it was more sensitive than CT scan of the chest. However early thoracic CT scan has been very important in detecting injuries missed out by chest x-ray. Neither ultrasound nor CT scan of the chest was done in our study.

The majority of our patients were managed by non-operative approach which is in agreement with other studies [16,29-37]. This study has demonstrated that the majority of patients presenting with chest injury without associated injuries can be managed with procedures which can be readily performed in rural hospitals by well trained junior surgeons or experienced general practitioners using simple equipment such as chest tubes and underwater seal bottles. Thoracic surgeons generally agree that most patients with especially penetrating chest injuries could be managed adequately by closed thoracostomy tube drainage alone. Inci *et al *[8] reported the percentage to be between 62.1% and 91.4%. Close monitoring of the bluntly injured patient is of paramount with repeated examination, radiographs, aortography, electrocardiogram, and CT scan of the chest and blood gas analysis as appropriate to detect changes.

The presence of complications has an impact on the final outcome of patients presenting with chest injuries. The pattern of complications in the present study is similar to what was reported in Nigeria by Ali *et al *[15]. Early recognition and management of complications following chest injury is of paramount in reducing the morbidity and mortality resulting from chest injuries.

The overall mean LOS in this study was higher compared to that reported by Atri *et al *[28] in India, but lower than that reported in the Nigerian study [15]. Non-survivors in the present study were found to have a shorter mean LOS and majority of deaths occurred during the first 24 hours of admission. The long period of hospital stay in our study was noted in patients with penetrating chest injuries and those with associated head injuries and long bone fractures. The length of hospital stay is an important measure of morbidity. Estimates of length of hospital stay are important for financial reasons, and accurate early estimates facilitate better financial planning by the payers. The overall mortality rate in this study was 4.7% comparable to that found in Nigeria [15], but relatively lower than that reported in other studies [16,20,28,37]. The reason for low mortality rate in the present study is that most of the patients were not severely injured except when there was a major associated extra-thoracic injury. They responded favorably to measures that were well within the competence of general surgeons and registrars. Limited study period and unavailability of thoracostomy tubes were the major limitation in this study.

## Conclusions

Chest trauma is an important public health problem accounting for a substantial proportion of all trauma admissions at Bugando Medical Centre. The pattern of chest trauma and its management was almost similar to many series. RTC continues to be the major etiological factor for chest injuries and the commonly affected victims are young adult males in their productive and reproductive age group. Urgent preventive measures targeting at reducing the occurrence of RTCs is necessary to reduce the incidence of chest injuries in this region.

## Competing interests

The authors declare they have no competing interest.

## Authors' contributions

MKL contributed in study design, literature search, data analysis, manuscript writing & editing. PLC participated in study design, data analysis, manuscript writing & editing. JBM participated in data analysis, manuscript writing & editing. WM supervised the study and contributed in data analysis, manuscript writing & editing. All the authors read and approved the final manuscript.
